# Arrhythmias and Device Therapies in Cardiac Amyloidosis

**DOI:** 10.3390/jcm13051300

**Published:** 2024-02-25

**Authors:** Syed Bukhari, Syed Zamrak Khan, Mohamed Ghoweba, Bilal Khan, Zubair Bashir

**Affiliations:** 1 Department of Cardiovascular Medicine, Section of Vascular Medicine, Cleveland Clinic Foundation, Cleveland, OH 44195, USA; khans15@ccf.org (S.Z.K.); ghowebm@ccf.org (M.G.); 2 Department of Hospital Medicine, Temple University-Jeanes Campus, Philadelphia, PA 19111, USA; bilalmomand99@gmail.com; 3 Department of Hospital Medicine, Alpert Medical School of Brown University, Providence, RI 02903, USA; zubair_bashir@brown.edu

**Keywords:** cardiac amyloidosis, arrhythmia, left atrial appendage closure device, pacemaker, implantable cardioverter defibrillator

## Abstract

Cardiac amyloidosis is caused by amyloid fibrils that deposit in the myocardial interstitium, causing restrictive cardiomyopathy and eventually death. The electromechanical, inflammatory, and autonomic changes due to amyloid deposition result in arrhythmias. Atrial fibrillation is by far the most common arrhythmia. The rate control strategy is generally poorly tolerated due to restrictive filling physiology and heart rate dependance, favoring adoption of the rhythm control strategy. Anticoagulation for stroke prophylaxis is warranted, irrespective of CHA_2_DS_2_-VASc score in patients with a favorable bleeding profile; data on left appendage closure devices are still insufficient. Ventricular arrhythmias are also not uncommon, and the role of implantable cardioverter-defibrillator in cardiac amyloidosis is controversial. There is no evidence of improvement in outcomes when used for primary prevention in these patients. Bradyarrhythmia is most commonly associated with sudden cardiac death in cardiac amyloidosis. Pacemaker implantation can help provide symptomatic relief but does not confer mortality benefit.

## 1. Introduction

Cardiac amyloidosis (CA) is an underdiagnosed etiology of heart failure, which can lead to death if untreated. It results from myocardial deposition of insoluble amyloid fibrils that originate either from hepatically synthesized transthyretin protein (ATTR) or plasma cell-derived immunoglobulin light chain (AL) [1]. ATTR is further differentiated into wildtype (wtATTR) and hereditary (hATTR) based on the absence or presence of a mutation in the TTR gene sequence. While cardiac AL requires histological confirmation, ATTR can be diagnosed noninvasively with Tc-pyrophosphate scintigraphy in the majority of cases [2,3]. The clinical phenotype varies significantly between different types of CA, resulting in a spectrum of presentations.

AL is a hematological disorder caused by the proliferation of an abnormal plasma cell clone that overproduces lambda or kappa light chains. While it affects multiple organs, myocardial involvement has been reported in ~75% of the cases and determines prognosis [4]. The age at diagnosis is about 60–70 years, and it has a more aggressive disease course compared with ATTR due to cardiotoxicity associated with amyloidogenic light chains [4,5] These insoluble misfolded light chains exert cellular toxicity through various mechanisms, including disruption of tissue architecture and cellular membranes, induction of impaired calcium homeostasis, and the generation of reactive oxygen species, leading to cellular death [6,7].

Previously known as senile CA, wtATTR is being increasingly diagnosed with the advent of refined imaging modalities, and its prevalence ranges between 13% and 19% of patients hospitalized with heart failure with a preserved ejection fraction (HFpEF) [8,9]. Although left ventricular hypertrophy (LVH) is the most common echocardiographic feature in wtATTR, a prospective study found the prevalence of wtATTR in HFpEF patients without LVH (LV wall thickness <12 mm) to be ~5% [10]. The wtATTR typically affects elderly Caucasian men, and cardiac involvement is always present. On the other hand, hATTR demonstrates variability in the age of onset, primary phenotype (cardiomyopathy, neuropathy, or mixed), and disease course, depending on the mutation and fibril type. V122I mutation is the most common mutation in the US, affecting ~3–4% of African-American patients, and manifests predominantly with cardiomyopathy and heart failure [11]. V122I carriers are also at an increased risk of polyneuropathy, and the prevalence ranges between 2% and 9% [12]. The hATTR has been associated with increased all-cause mortality compared to wtATTR, with the poorest survival observed in V122I genotype [13]. V30M is the most common mutation world-wide and is found endemically in the northern parts of Sweden and in regions in Portugal and Japan [14]. V30M mutation manifests as a mixed phenotype, ranging from predominant neuropathy to exclusive cardiomyopathy. This phenotypic diversity is deemed to be related to biochemical differences in the composition of TTR fibrils [15].

**Figure 1:**
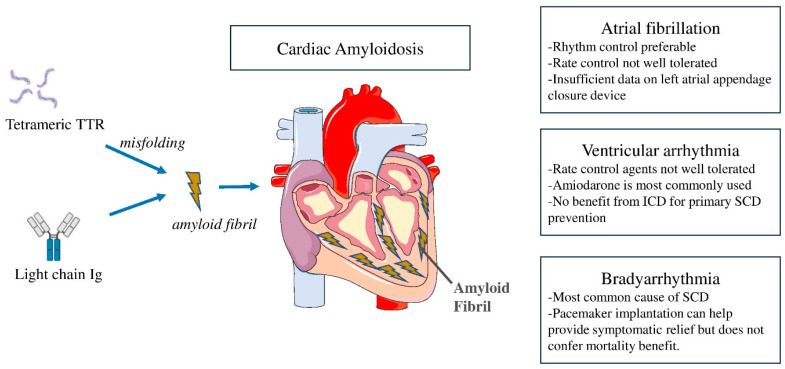
**Central illustration** Arrhythmias in cardiac amyloidosis and the role of cardiac devices. Ig = immunoglobulin; TTR = transthyretin; SCD = sudden cardiac death; ICD = implantable cardioverter-defibrillator.

Rhythm disturbances are a common occurrence in CA (Figure 1). The mechanisms are multifaceted, involving inflammatory triggers, structural changes with atrial dilatation, and neurohumoral abnormalities. While atrial tachyarrhythmias are most commonly seen in CA, ventricular tachyarrhythmias as well as bradyarrhythmias are also observed. The types of arrhythmias and their prevalence vary with CA subtypes. In this narrative review, we discuss mechanisms and epidemiology of various types of arrhythmias in CA, explore the utility of device therapies, and highlight knowledge gaps and future directions.

## 2. Atrial Arrhythmias in CA

### 2.1. Prevalence and Mechanisms

Atrial arrhythmias are more prevalent in CA compared with the general population. The prevalence of atrial fibrillation (AF) in CA has been reported to range from ~40% to 88% and is significantly higher in wtATTR compared with both AL and hATTR [16,17]. In a study of 238 patients examining all major CA subtypes (115 AL, 123 ATTR), the overall prevalence of AF was 44%, which is significantly higher than the prevalence of AF in the community [18,19,20]. Bukhari et al. reported that the prevalence of AF in wtATTR was two-fold higher compared to the age-matched, non-amyloid heart failure group (88% vs. 39%, *p* < 0.01) [17].

The development of AF In CA involves different mechanisms. The intramyocardial amyloid accumulation leads to impaired ventricular relaxation and elevated filling pressures, ultimately resulting in left atrial (LA) enlargement. In addition, amyloid deposition also disrupts electrical conduction and myocyte contractility leading to biomechanical dysfunction, which may provide an arrhythmogenic substrate for AF [21]. There can be isolated atrial amyloidosis from direct deposition of amyloid in atria, particularly in the LA, leading to impaired atrial mechanics [22,23,24]. Amyloid deposition in the atria disturbs myocyte contractility and disrupts homogenous electrical conduction, thereby providing an arrhythmogenic substrate for the development of AF by giving rise to functional re-entry [25]. It is thought that AF, in turn, potentiates further amyloid deposition, thereby leading to a vicious cycle [22].

### 2.2. Management of AF and Role of Left Appendage Closure Device

The high prevalence of AF in CA underscores the importance of arrhythmia surveillance and control strategies. However, the presence of restrictive myocardial mechanics with a low and relatively fixed stroke volume means that higher heart rates are often needed to maintain adequate cardiac output in CA, making rate control strategies challenging [26]. In addition, postural hypotension in the setting of dysautonomia and impaired vasomotor function is further exacerbated by the negative inotropic effects of medications like non-dihydropyridine calcium channel blockers [27]. The direct inotropic effect of calcium channel blockers is further enhanced by their increased binding to the amyloid fibrils, and therefore some authors argue against their use in CA [28]. Similarly, beta blockers may also be poorly tolerated, but low-dose beta-blockers may be better tolerated than calcium channel blockers and have shown mortality benefits in ATTR patients with LVEF ≤ 40% [29]. Amyloid fibrils also have an affinity for digoxin, but studies have shown that cautious use and frequent monitoring of serum drug levels can mitigate medication side effects [30,31,32].

Rhythm control, which improves ventricular filling through restoration of atrial function is potentially a more favorable and often well-tolerated strategy in CA [33]. Rhythm control strategy is more effective when introduced earlier during the disease course [34]. However, it is not associated with improved survival [17,34]. The data on the safety of antiarrhythmics are insufficient in this population. Many antiarrhythmic agents cannot be used in CA either due to cardiomyopathy (such as flecainide and propafenone) or due to concomitant advanced kidney disease (such as sotalol, dronedarone, and dofetilide). Amiodarone is the preferred agent and most commonly used in this population. Direct current cardioversion (DCCV) is a vital option for restoring sinus rhythm and potentially avoiding negative chronotropic agents. Its success rate is reported to be high and comparable to patients without CA [35]. However, the rate of complications and procedure cancellations is also high in comparison to non-CA patients, primarily due to a higher incidence of intracardiac thrombus identification [35]. Donnellan et al. reported restoration of sinus rhythm in 95% of patients, with sustained sinus rhythm observed in 45% at the 1-year follow-up, which was associated with a significantly lower mortality rate [34]. While DCCV may seem an appealing choice for restoring sinus rhythm, high procedural complications, higher risk of intracardiac thrombus identification, and an elevated risk of atrial arrhythmia recurrence should be considered in the decision-making process.

The data on the efficacy and safety of catheter ablation in CA are limited. The recurrence rate of atrial arrhythmia in CA patients undergoing ablation depends on the stage of the disease, ranging from 36% in stage 1 or 2 ATTR to 90% in stage 3 disease [34]. Tan et al. reported a 3-year arrhythmia-free survival in 40% of patients with AF, with 70% experiencing a reduction in the New York Heart Association functional class (NYHA-FC) after AF ablation [36]. Donnellan et al. reported a significant reduction in hospitalization for arrhythmia and heart failure and also a lower mortality rate in the AF ablation group compared with the non-ablation group [37]. Ablation was significantly more effective in those who were early in the disease course, as 64% of patients with stage I or II ATTR remained free of recurrent arrhythmia compared to only 10% patients with stage III disease (*p* = 0.005) [37]. Hence, AF ablation may be considered early in the disease course for patients who are refractory to antiarrhythmic therapy.

CA is associated with an increased risk of thromboembolism, and the presence of AF heightens this risk [18,38,39]). The CHA_2_DS_2_-VASc score has limited utility as a risk prediction tool. Anticoagulation is recommended in patients with CA and AF regardless of the CHA_2_DS_2_-VASc score [40]. Both direct oral anticoagulants (DOACs) and warfarin are comparable in terms of efficacy and safety profile in ATTR [41,42,43]. Bleeding risk should be assessed before initiation of anticoagulation in CA, although the lack of a standardized risk assessment tool can make decision making challenging. AL is associated with a higher risk of bleeding compared with ATTR. This is attributed to frequent involvement of the gastrointestinal tract, renal failure due to renal involvement, and the frequent presence of cytopenias either due to concomitant multiple myeloma or chemotherapeutic regimen [44]. Fortunately, most of these bleeding events are not major or fatal [44]. Mitrani et al. showed that there was no difference in thrombotic events and major bleeds between those who received warfarin and DOACs [41]. The thromboembolic event rate was 2.9/100 person years in the warfarin-treated group compared with 3.9/100 person years in the DOAC-treated group, while the event rate of major bleeds was 3.7/100 and 5.2/100 person years in the warfarin and DOAC groups, respectively [41]. Prospective studies are needed to definitively evaluate and compare the efficacy and safety of these anticoagulation strategies. In patients who are on anticoagulation, LA appendage thrombus can persist despite receiving therapeutic doses, making the anticoagulation strategy very challenging in CA [45]. The reported LA appendage thrombus resolution in the general population is approximately 90% after a median of 4 weeks of adequate anticoagulation, but the LA appendage thrombus resolution rate in CA is lower [45].

In patients who are intolerant to anticoagulation, the potential option is an LA appendage closure device, but it has not been studied extensively in CA yet. In the Cardiac Amyloidosis and Left Atrial Appendage Closure (CAMYLAAC) study, there were no significant differences in mortality between patients with and without CA (20% vs. 13.6%, *p* = 0.248) at the 2-year follow up. However, at the 5-year follow-up, ATTR patients had higher mortality (40% vs. 19.2%; *p* < 0.001), but importantly, this difference was unrelated to hemorrhagic complications or ischemic stroke [46]. Hence, while LA appendage closure in CA could provide an exciting opportunity for stroke prophylaxis when anticoagulation cannot be tolerated, prospective studies are required to evaluate its efficacy in CA.

## 3. Ventricular Tachyarrhythmias

### 3.1. Prevalence and Mechanisms

Ventricular tachyarrhythmias (VT) in CA could present as ventricular ectopy, non-sustained VT, sustained VT, or a combination of the above. The prevalence of VT in CA is higher than those without amyloidosis, and among the CA subtypes, it is higher in AL compared to ATTR. It has been reported to be 27% in AL as compared to 17% in ATTR patients [47,48]. AL patients undergoing stem cell transplantation have a higher prevalence of VT, likely due to the extent of myocardial damage caused by the aggressive nature of AL [49].

There are numerous mechanisms of pathogenesis of VT in CA, including electromechanical dysfunction and autonomic dysregulation that are uniquely related to systemic amyloid deposition [50]. Amyloid deposition results in the activation of the inflammatory cascade and oxidative stress, leading to ventricular remodeling and fibrosis [51,52]. This, in addition to microvascular ischemia caused by amyloid infiltration, results in the development of anatomical re-entrant circuits causing VT [53]. The pre-fibrillar amyloidogenic light chains are also more cytotoxic than ATTR, inducing a greater degree of apoptosis through oxidative stress, which potentially explains the higher prevalence of VT in AL compared with ATTR [4].

### 3.2. Management of VT and Role of Implantable Cardioverter-Defibrillator

CA patients poorly tolerate most of the medications that are used to control VT, including calcium channel blockers and beta-blockers. Amiodarone is most commonly used as an antiarrhythmic agent in CA. The role of catheter ablation has not been validated in large-scale studies.

CA, especially AL, is associated with an increased risk of SCD, accounting for approximately one-third of the mortality within the first 90 days of AL diagnosis [53]. Despite the high risk of SCD in CA, consensus guidelines have demonstrated little enthusiasm for implantable cardioverter-defibrillator (ICD) placement for the prevention of SCD in CA. The most frequent etiology of SCD in CA is pulseless electrical activity or bradyarrhythmia due to electromechanical dissociation, rather than VT, and therefore, it is important to identify the subset of patients that would get benefit from ICD placement [47,53]. ICD implantation in CA remains a grey zone that requires the adoption of an individualized, patient-specific approach [54,55].

Data on the efficacy of ICD for primary or secondary prevention in CA are scant and primarily derived from case reports and small-scale observational studies (Table 1) [56,57,58,59,60,61,62]. There has been no mortality benefit demonstrated in these studies. In a study comprised of 19 histologically proven AL patients who were followed for a mean period of 811 ± 151 days, two patients with sustained VT were successfully treated by the ICD. Seven (37%) patients died during the study period, and the majority (*n* = 6) died due to electromechanical dissociation not amenable to ICD treatment. The authors deduced that, while the majority of SCDs were caused by bradyarrhythmias that were not amenable to ICD, a select group of patients with life-threatening tachyarrhythmias could potentially benefit from ICD implantation.

ICD implantation specifically for primary prevention has no proven benefit and could even be harmful [59,60]. A single-center study examined 53 CA patients who underwent ICD implantation for either primary prevention of SCD (*n* = 41) or secondary prevention (*n* = 12) [59]. The rate of appropriate ICD shocks was 32% at the 1-year follow-up, observed almost exclusively in AL patients and those who had received an ICD for secondary prevention (*p* < 0.001); however, ICD therapy was not associated with improved mortality in the follow-up.

Another study from Stanford University revealed that 26% of 19 patients received appropriate ICD shocks in the follow-up, and 3 of 5 patients had received an ICD for secondary prevention [57]. Additionally, the authors proposed criteria for appropriate implantation in patients with CA, including patients who have a high risk of SCD as well as a good quality of life and minimal heart failure symptoms, as assessed by the NYHA-FC, and excluding patients who have a life expectancy of <1 year. The criteria proposed that CA patients who have a life expectancy of >1 year or NYHA-FC I-III could be candidates for ICD implantation if they have (1) non-postural exertional syncope and/or (2) evidence of NSVT or VT on telemetric monitoring. There is emerging data that extracellular volume on cardiac magnetic resonance can predict the incident ventricular arrhythmia, irrespective of the etiology of cardiomyopathy, thereby providing an exciting research avenue to identify CA patients who may benefit from ICD [63]. The 2022 European Society of Cardiology guidelines state that an ICD should be considered in ATTR or AL patients with hemodynamically not-tolerated VT after careful discussion of the competing risks of non-arrhythmic death and non-cardiac death (Class IIa, Level of Evidence: C) [64].

In summary, the decisions of ICD implantation should be cautiously considered and discussed between the patient and physician at an early stage, carefully weighing the risks and benefits of the procedure on life expectancy and quality of life.

### 3.3. Epidemiology of Bradyarrhythmias and Role of Pacemaker in CA

CA is commonly associated with bradyarrhythmias. Atrioventricular conduction delay involving the His-Purkinje system is more common than pure sinus node disease and is associated with symptomatic AV block [65]. A study comprised of 16 consecutive patients with hATTR with polyneuropathy who underwent 24 h ambulatory monitoring found evidence of sinus pauses (defined as >2 s) in 25% and conduction disturbances in 38% of the patients. During a follow-up period of 14 months, 5 patients received pacemaker implantation for either advanced AV nodal block or sinus node dysfunction [66]. In another cohort of 18 CA patients (4 AL, 14 wtATTR), who were analyzed using electrophysiological studies and compared with age- and gender-matched non-CA patients, CA patients were found to have significant prolongation of AH and HV intervals with a lesser degree of QRS prolongation. Notably, the prolongation was more profound in wtATTR compared with AL [65].

The prevalence of pacemaker implantation has been variably reported to range from 8.9% to ~40%, depending on the study size as well as the subtype of CA under investigation [67,68,69]. Patients with wtATTR have higher rates of pacemaker implantation compared with both AL and hATTR, which is attributed to the older age and less aggressive disease course in these patients. A history of AF, PR > 200 ms, and QRS > 120 ms on EKG are predictors of pacemaker placement in CA [67].

CA patients with pacemakers are noted to have progressive conduction disease and eventually become dependent on ventricular pacing. The use of cardiac implantable electronic devices (CIED) in CA patients has provided a window for the surveillance of rhythm disturbances and has given insight into the patterns of disease progression. In a single-center, retrospective study, longitudinal data was analyzed in 34 CA patients who had undergone CIED implantation for bradycardia or SCD. The interrogation of device data for 3.1 ± 4 years showed a progressive increase in the mean ventricular pacing, and the pacing burden increased from 56% at 1-year post-implantation to ~100% ventricular pacing at 5 years in the majority of patients [70,71].

The progressive Increase in the right ventricular pacing burden causes impairment of cardiac function and is associated with worse outcomes in CA. In a retrospective, observational cohort study of 78 patients with ATTR and implantable devices, RV pacing was associated with worsening ejection fraction, mitral regurgitation, and heart failure symptoms [72]. On the other hand, most patients met the standard criteria for cardiac resynchronization therapy, which resulted in improvement in the ejection fraction, systemic congestion, and mitral regurgitation severity [72]. In another retrospective study from the Cleveland Clinic, cardiac resynchronization therapy was associated with improved survival among patients with ATTR and also resulted in improvements in heart failure symptoms and the left ventricular ejection fraction [73].

In a study from UK National Amyloidosis Centre that used loop recorders to characterize arrhythmias in Mayo stage III AL patients, it was discovered that marked bradyarrhythmia heralded terminal cardiac decompensation in the majority of patients [74]. Over a median follow-up period of 308 days, 13 patients died, and the median survival in the whole cohort was 61 days from device insertion. In eight evaluable cases, death was heralded by complete atrioventricular block, followed shortly thereafter by pulseless electrical activity. Three out of four patients who received pacemakers had rapid cardiac decompensation and died. Despite 272 loop recordings, there was only one episode of non-sustained VT, which was preceded by severe bradycardia. The authors proposed that a study of prophylactic pacemaker implantation in this patient population is needed. While there are small-scale studies supporting the use of prophylactic pacemakers in hATTR, large-scale and multi-center studies are needed to assess the effectiveness of prophylactic pacemakers in all subtypes of CA [75,76].

The leadless pacemakers have not been extensively studied in CA. The use of ventricular transvenous pacemakers could potentially worsen tricuspid regurgitation in CA, as endocardial leads may cross the valve and impinge on thickened tricuspid leaflets. Leadless pacemakers may partially overcome this problem and can be a promising option. However, case reports have highlighted the risk of device dislocation, purportedly caused by instability and slippage of the device tines due to the deposition of amyloid between the myocytes [77].

### 3.4. Knowledge Gaps and Future Directions

The recognition of CA has grown significantly with the advent of non-invasive testing, contributing to an enhanced understanding of the disease and its associations, including arrhythmias [78,79]. A comprehensive, multi-center collaborative effort is crucial to define arrhythmias associated with CA, allowing for a better understanding of the arrhythmia burden, its mechanisms, and the associated clinical outcomes in this population. The current landscape lacks prospective and large-scale studies to validate the efficacy of devices such as the Watchman’s device, pacemakers, or ICDs in CA patients. It remains to be seen whether rhythm control with anti-arrhythmic agents, catheter ablation, or DCCV is superior to rate control for atrial tachyarrhythmias in these patients. Similarly, more studies are needed to assess the efficacy of early implantation of a pacemaker in patients with a high risk of heart block, which may also serve to monitor arrhythmias. Moreover, the utility of biventricular pacing and the role of ventricular synchrony in CA cardiomyopathy needs further clarification. Future investigations should focus on identifying clinical factors and imaging biomarkers to predict fatal arrhythmias in CA patients, aiming to identify a subset of patients that may derive a mortality benefit from cardiac devices.

There is a need for the development of validated risk assessment tools for thromboembolic and bleeding risk in CA. Mechanisms leading to exceptionally high thromboembolic risk in CA are yet unclear. In addition, no prospective, large-scale studies are available to compare the efficacy and safety profile of direct oral anticoagulants and Warfarin for the prevention of stroke and systemic embolism in CA patients with AF.

The disease-modifying drugs for CA act by preventing amyloid formation and deposition, but none target the removal of existing amyloid deposits from the affected organ. Removing pre-existing amyloid deposits to potentially restore organ function remains a huge treatment gap. A monoclonal antibody-based therapy is under evaluation that has the capability of selectively binding to and removing the pre-existing amyloid deposits. Such therapies have a tremendous potential to alter the course of this fatal disease [80]. It also remains to be seen whether advancements in therapies for both ATTR and AL have a favorable impact on the prevention of life-threatening arrhythmias and also on the mitigation of stroke risk in AF.

## 4. Conclusions

CA arises from the deposition of amyloid fibrils in the myocardial interstitium, triggering electromechanical, inflammatory, and autonomic changes that result in arrhythmias. A prevalent arrhythmia in this condition is atrial fibrillation, posing management challenges with insufficient data on left appendage closure devices. Ventricular arrhythmias are not uncommon, and the role of ICD in CA remains controversial, with no evidence of improved outcomes in primary prevention cases. The most common arrhythmias leading to sudden cardiac death are bradyarrhythmias and complete heart block. Although pacemaker implantation can provide symptomatic relief, it does not confer a mortality benefit.

## Figures and Tables

**Table 1:** Studies demonstrating use of ICD in CA.

Study	Population Demographics with ICD	ICD for Primary Prevention, N (%)	Successful ICD Therapy, %	Mean Age, Years	Mean Follow Up Duration, Months	1-Year Survival, %
Kristen et al. [56]	N = 19	19 (100)	11	58	27 ± 5.0	63
Varr et al. [57]	N = 19 AL-15 ATTR-4	15 (79)	26	68	NA	50
Hamon et al. [58]	N = 45 AL-12 ATTR-33	38 (84)	27	66	17 ± 14	73
Lin et al. [59]	N = 53 AL-33 ATTR-19	41 (77)	32	64	23 ± 21	22
Hiigins et al. [60]	N = 472	359 (76)	NA	68	NA	73
Donnellan et al. [61]	N = 19 ATTR-19	19 (100)	NA	73	23 ± 19	16
Brown et al. [62]	N = 32 ATTR-32	32 (100)	25	74	38 ± 3.6	75

ICD: Implantable cardioverter-defibrillator; CA: Cardiac amyloidosis; NA: not available.

## Data Availability

No research data created.
